# Dietary Macronutrient Intakes and Mortality among Patients with Type 2 Diabetes

**DOI:** 10.3390/nu12061665

**Published:** 2020-06-03

**Authors:** Cheng-Chieh Lin, Chiu-Shong Liu, Chia-Ing Li, Chih-Hsueh Lin, Wen-Yuan Lin, Mu-Cyun Wang, Shing-Yu Yang, Tsai-Chung Li

**Affiliations:** 1 School of Medicine, College of Medicine, China Medical University, Taichung 404, Taiwan; cclin@mail.cmuh.org.tw (C.-C.L.); liucs@ms14.hinet.net (C.-S.L.); t6446@mail.cmuh.org.tw (C.-I.L.); d5496@mail.cmuh.org.tw (C.-H.L.); wylin@mail.cmu.edu.tw (W.-Y.L.); d13067@mail.cmuh.org.tw (M.-C.W.); 2 Department of Family Medicine, China Medical University Hospital, Taichung 404, Taiwan; 3 Department of Medical Research, China Medical University Hospital, Taichung 404, Taiwan; 4 Department of Public Health, College of Public Health, China Medical University, Taichung 404, Taiwan; yz123kimo@yahoo.com.tw; 5 Department of Healthcare Administration, College of Medical and Health Science, Asia University, Taichung 413, Taiwan

**Keywords:** carbohydrate, protein, fat, type 2 diabetes, mortality

## Abstract

The best macronutrient percentages of dietary intake supporting longevity remains unclear. The strength of association between dietary intake and mortality in patients with type 2 diabetes (T2DM) should be quantified as a basis for dietary recommendations. Our study cohort consisted of 15,289 type 2 diabetic patients aged 30 years and older in Taiwan during 2001–2014 and was followed up through 2016. Percentages of macronutrient intakes were calculated as dietary energy intake contributed by carbohydrate, protein, and fat, divided by the total energy intake using a 24 h food diary recall approach. Cox proportional hazard models were applied to examine the temporal relation of macronutrient intakes with all-cause and cause-specific mortality. The average follow-up time was 7.4 years, during which 2784 adults with T2DM died. After multivariable adjustment, people with fourth and fifth quintiles of total energy, second and third quintiles of carbohydrate, and fourth quintiles of protein intakes were likely to have lower risks of all-cause and expanded cardiovascular disease (CVD) mortality. People with fifth quintiles of total energy intake were likely to have decreased non-expanded CVD mortality. We found a significant interaction between gender and fat intake on all-cause and expanded CVD mortality. Fat intake was associated with all-cause, expanded and non-expanded CVD mortality among males with T2DM. Total energy, carbohydrate, and protein intakes were associated with lower risks of all-cause and expanded CVD mortality, with minimal risks observed at ≥1673 Kcal total energy, 43–52% carbohydrate intake, and 15–16% protein intake among people with T2DM.

## 1. Introduction

The population with diabetes mellitus (DM) is growing annually globally, especially in the Asian region [1]. More than 5 million people worldwide died of diabetes in 2017, compared with less than 1 million people in 2000 [2]. The statistics report indicated that more than 60% of people with diabetes worldwide live in Asia and the prevalence of diabetes ranged from 3% to 47% in Asian countries [3]. The prevalence and incidence of diabetes have rapidly increased in Asia, due to westernized lifestyle behaviors and the increased prevalence of obesity [3,4]. DM and its associated complications are leading causes of work loss, disability, and premature mortality, reinforcing a heavy burden of medical, social, and economic costs [5,6].

Lifestyle modifications with anti-diabetes medications can prevent premature morbidity and mortality, by reducing the risks of diabetic microvascular and macrovascular diseases [7,8]. The most difficult work for people with diabetes is to achieve a balance between dietary desires and the compliance with behavior modification for disease management. Several studies have shown the relationship between dietary intake (carbohydrate, fat, and protein) and glycemic control [9,10,11] among people with diabetes. Reports indicated that hemoglobin A1c (HbA_1_c) level is negatively associated with carbohydrate [11] and protein [9] intakes and positively associated with fat [11] and total energy [10] intakes. People with diabetes and poor glycemic control are likely to have high mortality [12]. Although the effects of these individuals′ dietary behaviors on glucose control status have been explored [9,10,11], knowledge about the associations between individuals’ dietary behaviors and mortality in population with diabetes is minimal [12]. Understanding the associations of these modifiable factors on mortality in people with type 2 diabetes mellitus (T2DM) will have great clinical significance for diabetes care. The important contribution of this study is to provide information about an adequate and balanced dietary intake for nutritional modification for disease management, especially for people with T2DM.

Dietary components and nutritional strategies are fundamental in diabetes management. Previous studies have indicated that dietary components are associated with all-cause and disease-specific mortality [13,14,15,16,17,18,19,20,21,22,23,24,25,26] in various populations, most focusing on general populations. However, the results of these studies have been inconsistent; that is, some studies have shown positive [13,14,15,16,17,18,19,20,21], no [22,23], or negative [16,17,24,25,26] effects. In addition, most of these studies have focused on one dietary component, such as carbohydrate [13,15,21,22], protein [20,25], or fat or saturated lipids [14,18,19,23,26], and few studies have simultaneously considered these three dietary components [16,17]. To our knowledge, no study has assessed whether the three dietary macronutrient intakes are simultaneously associated with all-cause and cause-specific mortality among patients (focusing on the Chinese population) with T2DM. Furthermore, the percentages of carbohydrate, protein, and fat intakes associated with mortality among people with T2DM remain unclear. Consequently, we evaluated the link of total energy, carbohydrate, protein, and fat intakes with subsequent all-cause and disease-specific mortality among Chinese people with T2DM.

## 2. Materials and Methods

### 2.1. Study Subjects

**Figure 1:**
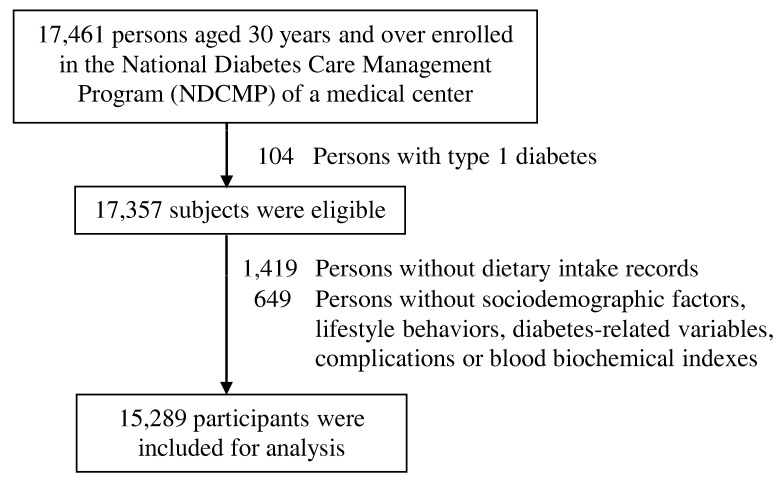
Flowchart of recruitment procedures for the current study.

A retrospective cohort study was conducted among patients with T2DM enrolled in the Diabetes Care Management Program (DCMP) of China Medical University Hospital (CMUH), which is a case management program set up by the National Health Insurance Administration in 2001. The goal of the DCMP is to improve diabetes care quality through intensive monitoring and the provision of continuous care for decreasing diabetes-related complications. Enrollees were people diagnosed with T2DM (International Classification of Diseases, Ninth Revision, Clinical Modification (ICD-9-CM); code 250). All eligible cases included those who were enrolled in the registry between November 2001 and December 2014 and who had to be continuously enrolled in the DCMP until December 2016, death, or withdrawal. Each subject entered the study at different time points with unequal follow-up intervals; therefore, this cohort is open or dynamic. Index date was defined as the date of entry into the DCMP. We excluded patients with type 1 (ICD-9-CM code 250.x1/x3, x= 0–9) and gestational (ICD-9-CM code 648.83) diabetes and people aging <30 years and without anthropometric measurements, laboratory examination, and dietary records. These criteria were met by 15,289 continuously enrolled people with T2DM (Figure 1). The Ethical Review Board of CMUH approved the study protocol (CMUH107-REC3-150).

### 2.2. Data Source

Data were obtained from the computerized database of people with T2DM enrolled in the DCMP of CMUH in Taichung, Taiwan. Under the DCMP, health care providers must receive clinical education and training programs for certification to be eligible health care providers of DCMP and enroll patients voluntarily into this program. The health care providers consist of physicians from endocrinology, nephrology, cardiology, internal medicine, family medicine, and other specializations. The continuing education and training programs promote the standardization of clinical practice, such as the assessment and diagnosis of diabetes-related complications. Physician-led multidisciplinary teams, including physicians, nurse case managers and dietitians, work together to provide coordinated care and help their patients adhere to established clinical guidelines. The DCMP database annually provides information, including self-care education and assessments, lifestyle behavior interventions, eye and foot examinations, and laboratory tests.

### 2.3. Measurements

People at the time of entering the DCMP had undergone a series of medical tests for blood, urine, and body measurements and answered questions on lifestyle, diet, and medical history with a standardized computerized questionnaire administered by a nurse case manager to record previous or current disease status. The variables are described as follows:

#### 2.3.1. Sociodemographic Factors, Lifestyle Factors, and Diabetes-Related Variables

The sociodemographic factors included age at baseline, gender, and family history of diabetes, hypertension, hyperlipidemia, and obesity. Lifestyle factors, such as smoking, alcohol drinking, and exercise, were each divided into two groups: yes and no. Diabetes-related variables included the early onset of T2DM, defined as age of diabetes onset of less than 40 years old, and the duration of T2DM. Types of diabetes treatment consisting of types of oral hypoglycemic agents (e.g., biguanide, sulfonylurea, thiazolidinedione, meglitinide, and metformin) and insulin therapy were extracted from the medical record.

#### 2.3.2. Anthropometric Measurements and Complications

Weight and height were measured using an auto-anthropometer (super-view, HW-666, Taipei, Taiwan), wherein the subjects were shoeless and wearing light clothing. Body mass index (BMI) was derived from the following formula: weight (kg)/height (m^2^). Blood pressure was measured three times using an electronic device (COLIN, VP-1000, Komaki, Japan).

Baseline diabetes-related comorbidities consisted of hypertension, hyperlipidemia, stroke, coronary artery disease, severe hypoglycemia, peripheral neuropathy, and nephropathy. These comorbidities are each divided into two groups: yes and no.

#### 2.3.3. Dietary Intake

A 24 h dietary recall (24HDR), which is a structured interview, was used to capture detailed quantitative information about all foods, beverages, and dietary supplements consumed by the study subjects in the past 24 h, from midnight of the present day to midnight of the previous day. The type of question in 24HDR was an open-ended response structure, which was used to ask respondents about the type and quantity of all foods and beverages consumed. For example, a respondent reporting a sandwich for lunch was asked about the preparation method and bread type. In addition, time of day, source of food, portion size or common unit, and other attributes of each food and beverage were reported. Food models, pictures, and other visual aids were used to improve the quality of data reported by respondents. Dietary recalls ask about foods and beverages first before questions on dietary supplements. A 24HDR often requires 20–60 min to complete. The 24HDRs were administered by a trained clinical dietitians. The food data of a single 24HDR were matched with nutrient information from a food composition database to analyze the macronutrient content. The energy and macronutrient intakes from each food item were then derived. The daily energy intake was calculated by multiplying the amount of consumption of each item by its caloric content per serving and then totaling the caloric intake for all food items. The percentage of total kilocalories from carbohydrate, protein, and fat intakes was then derived.

#### 2.3.4. Laboratory Examination

Blood was drawn from an antecubital vein in the morning after a 12 h overnight fasting and was sent for analysis within 4 h of blood collection. Biochemical markers, such as high-density lipoprotein (HDL), low-density lipoprotein (LDL), and fasting plasma glucose (FPG), were analyzed with a biochemical auto-analyzer (Beckman Coulter Synchron system, Lx-20, Fullerton, CA, USA), at the Clinical Laboratory Department of CMUH. FPG was measured in blood obtained using a sodium fluoride (NaF) tube, which contained 5 mg of sodium fluoride to inhibit glucose metabolism and 4 mg of potassium oxalate to chelate calcium and prevent coagulation. Inter- and intra-assay coefficients of variations (CVs) for FPG were both 4%. HbA1c level was measured using a Boronate affinity high-performance liquid chromatography assay (reference range, 4.6–6.5%). The inter- and intra-assay CVs for HbA1c were 2.91% for the normal level, 1.79% for the intermediate level, and 1.09% for the high level. HDL and LDL levels were measured via a direct method; and the inter- and intra-assay CVs for HDL were both 4.5% and for LDL were 4.5% and 3%, respectively.

Annual measurements of HbA1c for each person in the electronic records were retrieved and analyzed. HbA1c is a form of hemoglobin A that results from the non-enzymatic attachment of glucose to hemoglobin A. The percentage of HbA1C depends on the average glucose concentration over the life of a red blood cell (120 days), making HbA1C a good measure of glycemic control over the preceding 2–3 months. HbA1c is at the center of the clinical management of hyperglycemia and is considered an important monitoring tool in treating people with diabetes. HbA1c has been treated as a better long-term glycemic control marker than fasting glucose, because it can be tested in a non-fasting status and is a relatively stable marker for glucose level.

#### 2.3.5. Outcome Measures

Main outcome measures included all-cause mortality and expanded and non-expanded cardiovascular disease (CVD)-related mortality. Such measures were determined through an annual record linkage with the National Death Datasets, by using personal identification number and date of birth provided by the Taiwan Ministry of Health and Welfare. The people were followed up from the index date to 31 December 2016, or until death or withdrawal from the DCMP. The Cause of Death Registry was coded in accordance with the rules of the ICD-9-CM 2006–2008 and the International Classification of Diseases, 10th Revision, Clinical Modification (ICD-10-CM) 2009–2016. Deaths were classified as expanded and non-expanded CVD-related diseases. Expanded CVD mortality was defined as death due to CVD (ICD-9-CM codes 390–459, ICD-10-CM codes I00–I99), diabetes (ICD-9-CM code 250, ICD-10-CM codes E10–E14), and kidney diseases (ICD-9-CM 580–589; ICD-10-CM N00–N29). Non-expanded CVD mortality included all causes, excluding expanded CVD causes.

### 2.4. Statistical Analysis

Baseline characteristics were presented as frequency (percentage) by using the Chi-square tests for categorical variables and as means ± standard deviations (SDs) by using the *t*-tests for continuous variables. Study participants were classified into subgroups on the basis of quintiles of macronutrient intakes, such as total energy, carbohydrate, fat, and protein. In the survival analyses of macronutrient intakes and mortality risks, survival curves were estimated using the Kaplan—Meier method, and the differences in the entire survival functions among groups were determined using log-rank tests. Cox proportional hazard models were applied to examine the relationship of total energy, carbohydrate, protein, and fat intakes with mortality. Hazard ratios (HRs) and their 95% confidence intervals (CIs) were presented after adjusting for age, gender, lifestyle behaviors, diabetes-related, and multiple traditional variables. Interaction terms of gender and macronutrient intakes were assessed to determine whether gender is an effect modifier on the relationships between macronutrient intakes and mortality. The dose-response relationships were assessed using linear trend test across the quintile subgroups. Restricted cubic splines were applied in Cox models to examine the dose—response or nonlinear association of macronutrient intake as a continuous variable with mortality. All analyses were performed using SAS version 9.4 (SAS, Cary, NC, USA). All *p* values were two-tailed, and a *p* value <0.05 was considered statistically significant.

## 3. Results

A total of 15,289 individuals with a mean follow-up of 7.4 years were included, and 2784 deaths were recorded (1287 expanded and 1497 non-expanded CVD deaths), with a crude mortality rate of 24.7/1000 person-years (11.4 for expanded and 13.3 for non-expanded CVD mortality). As shown in Table 1, people who died during the follow-up period were associated with several variables: old age, male, smoking, low BMI, long diabetes duration, and insulin use; history of hypertension, hyperlipidemia, stroke, coronary artery disease, severe hypoglycemia, peripheral neuropathy, and nephropathy; low levels of total energy intake, fat intake, and HDL; and high levels of carbohydrate intake, HbA1c, and fasting plasma glucose.

Table 2 presents the macronutrient intake and risks of all-cause, expanded CVD, and non-expanded CVD mortality. A high level of total energy intake was negatively correlated with all-cause mortality (quintile 4 vs. quintile 1, HR: 0.85 [95% CI: 0.75–0.96]; quintile 5 vs. quintile 1, 0.80 [0.70–0.91]; *p* for trend <0.001), expanded CVD mortality (quintile 4 vs. quintile 1, 0.81 [0.67–0.97]; *p* for trend = 0.02), and non-expanded CVD mortality (quintile 5 vs. quintile 1, 0.78 [0.65–0.94]; *p* for trend = 0.003). In comparison with quintile 1, quintiles 2 and 3 of carbohydrate intake were inversely associated with risks of all-cause mortality (0.86 [0.76–0.97] and 0.85 [0.75–0.96], respectively; *p* for trend = 0.63) and expanded CVD mortality (0.78 [0.65–0.94] and 0.83 [0.70–0.99], respectively; *p* for trend = 0.79). In comparison with quintile 1, quintile 4 of protein intake was inversely associated with risks of all-cause mortality (0.85 [0.77–0.95]; *p* for trend = 0.008) and expanded CVD mortality (0.80 [0.69–0.94]; *p* for trend = 0.02).

**Figure 2:**
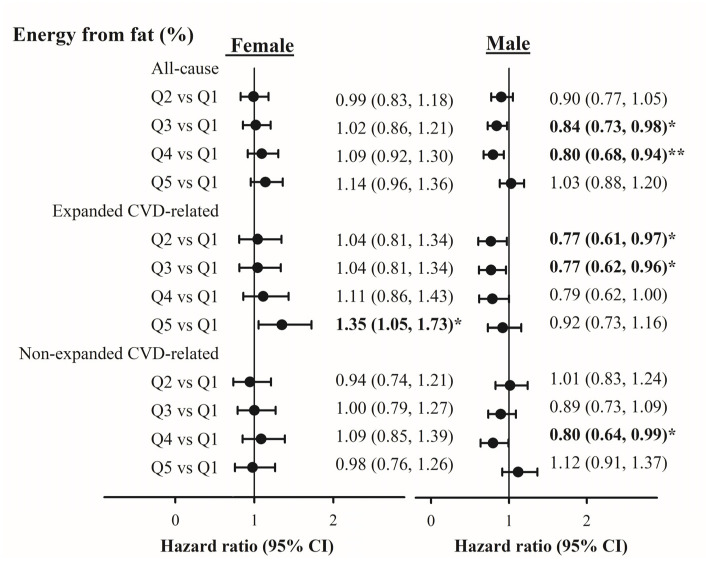
Associations between energy from fat and with risk of all-cause, expanded cardiovascular disease (CVD)-related, and non-expanded CVD-related mortality. Total Calcium Intake (Kcal): Quintile 1: <1210; Quintile 2: 1210–1454; Quintile 3: 1455–1672; Quintile 4: 1673–1964; Quintile 5: ≥1965. Energy from carbohydrate (%): Quintile 1: <43; Quintile 2: 43–47; Quintile 3: 48–52; Quintile 4: 53–57; Quintile 5: ≥58. Energy from fat (%): Quintile 1: <27; Quintile 2: 27–30; Quintile 3: 31–34; Quintile 4: 35–38; Quintile 5: ≥39. Energy from protein (%): Quintile 1: <13; Quintile 2: 13; Quintile 3: 14; Quintile 4: 15–16; Quintile 5: ≥16. CI: confidence interval; *: *p* < 0.05; **: *p* < 0.01. Bold values denote statistical significance at the *p* < 0.05 level.

Insignificant associations between fat intake and all-cause and cause-specific mortality were observed. Furthermore, significant interactions between gender and fat intake on all-cause and CVD-expanded mortality were found. Figure 2 shows the risks of mortality for different quintiles of fat intake for men and women. In men, HRs (95% CI) across quintile groups of fat intake were 1.00, 0.90 (0.77–1.05), 0.84 (0.73–0.98), 0.80 (0.68–0.94), and 1.03 (0.88–1.20) for all-cause mortality (*p* for trend = 0.63); 1.00, 0.77 (0.61–0.97), 0.77 (0.62–0.96), 0.79 (0.62–1.00), and 0.92 (0.73–1.16) for expanded CVD mortality (*p* for trend = 0.43); and 1.00, 1.01 (0.83–1.24), 0.89 (0.73–1.09), 0.80 (0.64–0.99), and 1.12 (0.91–1.37) for non-expanded CVD mortality (*p* for trend = 0.99). Among women, expanded CVD mortality was positively associated with fat intake (quintile 5 vs. quintile 1, 1.35 [1.05–1.73]; *p* for trend = 0.02). A U-shaped association was observed between fat intake and mortality in men, but not in women.

**Figure 3:**
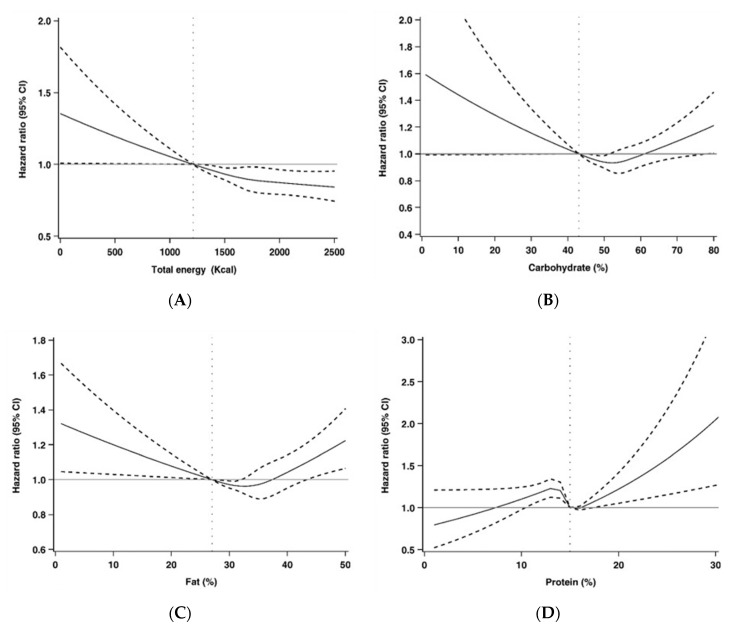
Restricted multivariable cubic spline plots for (**A**) total energy, (**B**) carbohydrate, (**C**) fat and (**D**) protein and all-cause mortality. CI: confidence interval.

Figure 3 shows the restricted multivariable cubic spline plots for all-cause mortality by total energy, carbohydrate, fat, and protein intakes. The X axis presents the possible values of total energy, carbohydrate, fat, and protein intakes and Y axis illustrates the hazard ratios and their 95% confidence intervals of all-cause mortality. The multivariable splines for carbohydrate, fat, and protein intakes demonstrated nonlinear associations with all-cause mortality.

## 4. Discussion

This work was a retrospective cohort study consisting of 15,289 people with T2DM from 2001 to 2014 and were then followed up until 2016 in Taiwan. Individuals with energy from 43–52% carbohydrate intake had a 14–15% lower risk of all-cause mortality and a 17–22% lower risk of expanded CVD mortality. We observed a 15% lower risk of all-cause mortality and 20% lower risk of expanded CVD mortality for energy from 15–16% protein intake, among patients with T2DM. In addition, women with ≥39% fat intake was associated with a 35% higher risk of expanded CVD mortality, whereas men with 27–30% fat intake was associated with a 23% lower risk of expanded CVD mortality. Men with 31–34% fat intake was associated with 16% and 23% low risks of all-cause and expanded CVD mortality, respectively. Men with 35–38% fat intake was associated with 20% lower risks of all-cause and non-expanded CVD mortality. Our results would provide useful information for the dietary/nutritional recommendations for the Chinese population with T2DM, to identify the optimal diets with the greatest effect on mortality, which is a public health priority.

DM is a metabolic disease resulting from defects in insulin secretion and/or insulin action. These defects cause hyperglycemia, with abnormalities in carbohydrate, fat, and protein metabolism [27]. Few epidemiologic studies observed the associations of carbohydrate, protein, and fat intake with the mortality of patients with diabetes [15,19]. Two studies from the European Prospective Investigation into cancer and nutrition cohort have supported the relation of carbohydrate and fat intakes with all-cause mortality among patients with diabetes [15,19]. Burger KN et al. reported that one standard deviation increase in carbohydrate intake is associated with a 67% increase in total mortality among normal-weight adults with diabetes, but not among diabetic and overweight adults [15]. Trichopoulou A et al. observed that one standard deviation increase in daily intake of saturated lipids is linked with an 82% increase in total mortality among adult diabetic patients [19]. Our study demonstrated that people with 43–52% carbohydrate intakes were associated with 17–22% decrease in all-cause and expanded CVD mortality, which was consistent with the findings of a previous meta-analysis [28]. The difference between findings of prior studies and ours is probably due to the differences in participants and the method of data collection for macronutrient intakes. In Burger’s study, country-specific questionnaires or food frequency questionnaires were used to collect dietary information; in Trichopoulou’s study, dietary intake was derived from food frequency questionnaires; and in our study, macronutrient intakes were derived from a 24 h dietary recall administered by a trained clinical dietitians.

Prior studies investigating the associations of fat and protein intakes on mortality have been controversial. A UK study found a significantly positive relationship between dietary total fat and CHD death in women, but not in men [14]. On the contrary, a Spain study reported an inverse relationship of total fat consumption with total and CVD mortality in people at high risk of CVD [26]. Two Japan studies supported the gender difference in the effects of fat intake on mortality [23,24]. One study indicated a U-shaped association between total fat intake and all-cause mortality in women, but not in men [23]; whereas another study observed an inverse association between total fat and mortality in men, but not in women [24]. Our study showed gender differences in the association between fat intake and mortality. Women had a positively increasing association between fat intake and expanded CVD mortality, whereas men had a U-shaped association for all-cause and expanded and non-expanded CVD-related mortality. Previous studies also found conflicting results on the relationships between protein intake and mortality [20,25]. As for the protein intake, an eastern Finnish study indicated that a high total protein intake is related to increased mortality risk, especially in people with T2DM, CVD, or cancer [20]. However, a France study reported an inverse association between protein intake and mortality in people with hypertension but without chronic kidney disease [25]. These two studies were conducted in the general population with subgroup analysis or people with hypertension, to explore this association among people with predisposing diseases, whereas our study focused on a cohort of people with T2DM and showed that individuals with T2DM who had 15–16% protein intake had the lowest all-cause and expanded CVD mortality. The differences in these studies’ findings may be due to the differences in food consumption patterns (foods, food groups, and included nutrients), accessibility to foods, food supply and process, agriculture in different regions, and cooking approaches, which may result in many differences in dietary patterns between eastern and western populations. In addition, the distributions of dietary components may vary dramatically across ethnic populations. As such, studies on fat intake and mortality conducted in populations having diet rich in monounsaturated and polyunsaturated fatty acids may have findings which contrast to those conducted in populations with diets rich in saturated fatty acid and trans-fat. Thus, the optimal dietary macronutrient intakes specific for the Chinese population with diabetes must be evaluated.

The strengths of our study include a large number of people with T2DM as subjects, the use of a standardized procedure for data collection (24HDR), detailed data on potential confounders, a high follow-up rate, and a long follow-up period. In addition, we adjusted for a great number of potential confounding factors, including glycemic control, diabetes duration, and types of diabetes treatment, in the final model.

Several limitations should be considered. First, confounding variables were measured at baseline; whether covariate changes happened over time during the follow-up period was unknown. Prior longitudinal studies exploring dietary patterns at the individual level over time found that the percentage of energy intake from fat decreases and that from carbohydrates and polyunsaturated or saturated fatty acid ratio increases as people get older [29,30]. As the dietary patterns did not change systematically in different categories of dietary macronutrient intakes, the non-differential error would not invalidate our findings. Second, measurement errors in self-reporting dietary and lifestyle behaviors were inevitable. To reduce such errors, participants completed a computerized, standardized questionnaire administered by case management nurses and dietitians to record their disease status, medication, lifestyle factors, and diet information. Third, the dietary data collected by 24HDR may have potential errors due to day-to-day variations arising from within-subject random error, which can be minimized if we measured dietary intake by using data for a small number of days. A single 24HDR was taken in the present study, due to limited time available in clinical practice. This type of within-person random error may lessen the power of the present study to detect the relationships between dietary intake and mortality, which causes a minor threat to our study findings. Fourth, the fat type, such as monounsaturated fat, polyunsaturated fatty acid, saturated fatty acid, and trans-fat, was not considered in this study. Thus, our findings on fat intake may only be valid when categories of energy from fat had balanced distributions in fat types, i.e., people in categories of energy from fat had similar food consumption patterns. Lastly, our study was hospital-based, not population-based; therefore, the entire population with T2DM in Taiwan may not be well represented. Such a selection bias may limit our findings to be generalized into other populations with diabetes, but they may be generalizable into other Chinese populations with diabetes, having similar characteristics to our study sample.

## 5. Conclusions

The top quintile of macronutrient intake (carbohydrate, fat, and protein) did not appear to have unfavorable effects on all-cause and cause-specific mortality among people with T2DM, except fat intake on expanded CVD-related mortality in women. Moderate carbohydrate (43–52% of energy) and high protein (15–16%) intakes contributed to a decrease in mortality. These results have important implications in establishing ethnic-specific dietary guidelines for Chinese people with T2DM.

## Figures and Tables

**Table 1:** The comparisons of baseline sociodemographic factors, lifestyle behaviors, diabetes-related variables, dietary intake, complications and blood biochemical indexes, according to mortality status in patients with type 2 diabetes enrolled in Diabetes Care Management Program of a medical center (*n* = 15,289).

Variables	Mortality Status *n* (%)		
	Alive (*n* = 12,505)	Dead (*n* = 2784)	*p* Value
**Sociodemographic Factors**			
Gender			<0.001
Female	6077 (48.6)	1219 (43.79)	
Male	6428 (51.4)	1565 (56.21)	
Age (years)	57.66 (11.57)	66.60 (10.95)	<0.001
**Life Style Behaviors**			
Smoking	2197 (17.57)	552 (19.83)	0.005
Alcohol Drinking	1064 (8.51)	242 (8.69)	0.78
Exercising	6419 (51.33)	1465 (52.62)	0.23
BMI (kg/m^2^)			<0.001
>25	5569 (44.53)	1476 (53.02)	
25–30	5123 (40.97)	1053 (37.82)	
≥30	1813 (14.50)	255 (9.16)	
**Dietary Intake†**			
Total Energy Intake (Kcal)	1611.30 (515.30)	1574.20 (473.30)	<0.001
Energy from Carbohydrate (%)	50.51 (9.61)	51.15 (9.65)	0.002
Energy from Fat (%)	32.76 (8.62)	32.08 (8.76)	<0.001
Energy from Protein (%)	14.64 (2.67)	14.66 (2.64)	0.81
**Diabetes-Related Variables**			
Duration of Diabetes (years) †	7.16 (7.22)	10.19 (8.79)	<0.001
Types of Diabetes Treatment			<0.001
Oral Agent Use Alone	10418 (83.31)	2207 (79.27)	
Insulin Injection Alone	166 (1.33)	48 (1.72)	
Oral Agent Plus Insulin Injection	1092 (8.73)	436 (15.66)	
Diet or Exercise	829 (6.63)	93 (3.34)	
**Complications**			
Hypertension	3835 (30.67)	1334 (47.92)	<0.001
Hyperlipidemia	2572 (20.57)	775 (27.84)	<0.001
Stroke	488 (3.90)	375 (13.47)	<0.001
Coronary Artery Disease	586 (4.69)	299 (10.74)	<0.001
Severe Hypoglycemia	91 (0.73)	79 (2.84)	<0.001
Peripheral Neuropathy	1084 (8.67)	588 (21.12)	<0.001
Nephropathy	464 (3.71)	320 (11.49)	<0.001
**Blood Biochemical Indexes †**			
High-Density Lipoprotein (mg/dL)	43.23 (11.50)	41.37 (12.77)	<0.001
Low-Density Lipoprotein (mg/dL)	113.20 (34.81)	113.60 (38.51)	0.67
HbA1c (%)	7.93 (1.77)	8.30 (1.98)	<0.001
Fasting Plasma Glucose (mg/dL)	152.30 (53.75)	165.90 (69.96)	<0.001

†: Mean (standard deviation). Student′s *t*-test was used for continuous variables to calculate *p*-values. Chi-square test was used for categorical variables to calculate *p*-values.

**Table 2:** The hazard ratios (HRs) of mortality, according to quintile of dietary intake in diabetic patients enrolled in Diabetes Care Management Program of a medical center (*n* = 15,289).

Variables	Age and Sex-Adjusted HR (95%CI)					*p* for Trend	Multivariable-Adjusted HR (95%CI)					*p* for Trend
	Q1	Q2	Q3	Q4	Q5		Q1	Q2	Q3	Q4	Q5	
**Total Energy Intake (Kcal)**												
All-Cause Mortality	1.00	0.94 (0.84, 1.06)	0.85 ** **(0.75, 0.95)**	0.84 ** **(0.74, 0.95)**	0.81 ** **(0.71, 0.92)**	<0.001	1.00	0.97 (0.86, 1.09)	0.89 (0.79, 1.00)	0.85 ** **(0.75, 0.96)**	0.80 *** **(0.70, 0.91)**	<0.001
Expanded CVD Mortality	1.00	0.90 (0.76, 1.07)	0.86 (0.72, 1.02)	0.81 * **(0.68, 0.97)**	0.83 (0.68, 1.01)	0.02	1.00	0.93 (0.78, 1.10)	0.90 (0.76, 1.07)	0.81 * **(0.67, 0.97)**	0.83 (0.68, 1.01)	0.02
Non-Expanded CVD Mortality	1.00	0.98 (0.83, 1.15)	0.84 * **(0.71, 0.99)**	0.87 (0.73, 1.02)	0.79 * **(0.66, 0.95)**	0.005	1.00	1.01 (0.86, 1.19)	0.88 (0.75, 1.04)	0.88 (0.75, 1.05)	0.78 ** **(0.65, 0.94)**	0.003
**Energy from Carbohydrate (%)**												
All-Cause Mortality	1.00	0.85 ** **(0.75, 0.96)**	0.81 *** **(0.72, 0.92)**	0.91 (0.81, 1.02)	0.91 (0.80, 1.02)	0.47	1.00	0.86 * **(0.76, 0.97)**	0.85 ** **(0.75, 0.96)**	0.91 (0.81, 1.03)	0.93 (0.82, 1.05)	0.63
Expanded CVD Mortality	1.00	0.77 ** **(0.64, 0.93)**	0.78* * **(0.66, 0.93)**	0.88 (0.74, 1.05)	0.88 (0.73, 1.04)	0.60	1.00	0.78 ** **(0.65, 0.94)**	0.83 * **(0.70, 0.99)**	0.87 (0.73, 1.04)	0.91 0.76, 1.09)	0.79
Non-Expanded CVD Mortality	1.00	0.92 (0.78, 1.08)	0.84 * **(0.72, 0.99)**	0.93 (0.79, 1.10)	0.94 (0.79, 1.11)	0.61	1.00	0.93 (0.79, 1.10)	0.86 (0.73, 1.02)	0.94 (0.80, 1.11)	0.94 (0.80, 1.12)	0.65
**Energy from Fat (%)**												
All-Cause Mortality	1.00	0.91 (0.81, 1.02)	0.92 (0.82, 1.02)	0.91 (0.81, 1.03)	1.09 (0.97, 1.22)	0.27	1.00	0.94 (0.84, 1.06)	0.91 (0.82, 1.02)	0.92 (0.82, 1.04)	1.08 (0.96, 1.21)	0.44
Expanded CVD Mortality	1.00	0.87 (0.73, 1.03)	0.89 (0.75, 1.05)	0.92 (0.78, 1.10)	1.12 (0.95, 1.32)	0.21	1.00	0.88 (0.75, 1.05)	0.88 (0.75, 1.04)	0.93 (0.78, 1.10)	1.09 (0.92, 1.29)	0.36
Non-Expanded CVD Mortality	1.00	0.94 (0.81, 1.10)	0.94 (0.81, 1.09)	0.90 (0.77, 1.06)	1.06 (0.91, 1.24)	0.75	1.00	0.99 (0.84, 1.16)	0.94 (0.81, 1.10)	0.92 (0.78, 1.08)	1.07 (0.91, 1.25)	0.81
**Energy from Protein (%)**												
All-Cause Mortality	1.00	1.05 (0.93, 1.19)	0.97 (0.87, 1.10)	0.86 ** **(0.78, 0.96)**	0.94 (0.83, 1.06)	0.01	1.00	1.04 (0.92, 1.18)	0.98 (0.87, 1.11)	0.85 ** **(0.77, 0.95)**	0.94 (0.83, 1.06)	0.008
Expanded CVD Mortality	1.00	0.98 (0.81, 1.17)	0.93 (0.79, 1.11)	0.82 * **(0.70, 0.96)**	0.90 (0.76, 1.07)	0.03	1.00	0.97 (0.81, 1.17)	0.94 (0.79, 1.12)	0.80 ** **(0.69, 0.94)**	0.90 (0.76, 1.08)	0.02
Non-Expanded CVD Mortality	1.00	1.12 (0.95, 1.33)	1.01 (0.86, 1.19)	0.90 (0.78, 1.04)	0.97 (0.83, 1.15)	0.13	1.00	1.11 (0.94, 1.31)	1.02 (0.87, 1.20)	0.90 (0.78, 1.04)	0.98 (0.83, 1.15)	0.15

Adjusted for sociodemographic factors, lifestyle behaviors, diabetes-related variables, blood biochemical indexes and complications. CVD: cardiovascular disease; HR: hazard ratio; CI: confidence interval; *: *p* < 0.05; **: *p* < 0.01; ***: *p* < 0.001. Total energy Intake (Kcal): Quintile 1: <1210; Quintile 2: 1210-1454; Quintile 3: 1455–1672; Quintile 4: 1673–1964; Quintile 5: ≥1965. Energy from carbohydrate (%): Quintile 1: <43; Quintile 2: 43–47; Quintile 3: 48–52; Quintile 4: 53–57; Quintile 5: ≥58. Energy from fat (%): Quintile 1: <27; Quintile 2: 27–30; Quintile 3: 31–34; Quintile 4: 35–38; Quintile 5: ≥39. Energy from protein (%): Quintile 1: <13; Quintile 2: 13; Quintile 3: 14; Quintile 4: 15; Quintile 5: ≥16. Bold values denote statistical significance at the *p* < 0.05 level.
